# Vascular Patterns in Retinitis Pigmentosa on Swept-Source Optical Coherence Tomography Angiography

**DOI:** 10.3390/jcm8091425

**Published:** 2019-09-10

**Authors:** Alessandro Arrigo, Francesco Romano, Giorgia Albertini, Emanuela Aragona, Francesco Bandello, Maurizio Battaglia Parodi

**Affiliations:** 1 Department of Ophthalmology, IRCCS Ospedale San Raffaele, University Vita-Salute, via Olgettina 60, 20132 Milan, Italy; 2 Eye Clinic, Department of Biomedical and Clinical Science, Luigi Sacco University Hospital, 20157 Milan, Italy

**Keywords:** Retinitis Pigmentosa, OCT, OCTA, vessel density, vessel tortuosity, vessel dispersion, vessel rarefaction

## Abstract

Background: Retinitis Pigmentosa (RP) represents a retinal dystrophy with an extremely complex pathogenesis further worsened by the impairment of the retinal vascular supply. The main goal of this study was to identify different vascular patterns in RP, by means of optical coherence tomography angiography (OCTA). Methods: A total of 32 RP patients (16 males, 50%; mean age 45.93 ± 11.4) and 32 healthy age-matched controls (16 males, 50%; age 42.8 ± 11.2). High resolution OCT and OCTA images were obtained from all participants. Several quantitative parameters were extracted both from structural OCT and OCTA images. A post-hoc analysis assessed the relationship between the quantitative OCTA parameters adopted and the following measures: best corrected visual acuity (BCVA), central macular thickness (CMT) and retinal nerve fiber layer (RNFL). Results: Mean LogMAR BCVA was 0.24 ± 0.32 for RP patients and 0.0 ± 0.0 for controls (*p* < 0.01). CMT, choroidal thickness and RNFL were statistically different between RP and controls (*p* < 0.01). OCTA parameters showed strong alterations of the retinal vascular network in RP (all *p* < 0.01). Several statistically significant correlations were also found. Furthermore, a vessel tortuosity cut-off of 4.80 and a vessel rarefaction cut-off of 0.62 enabled the RP cohort to be divided into two significantly different sub-groups in terms of BCVA, RNFL and CMT. Conclusions: Quantitative OCTA parameters help identify vascular abnormalities in RP, separating two different vascular patterns.

## 1. Introduction

The term Retinitis Pigmentosa (RP) covers a heterogeneous group of retinal disorders clinically characterized by progressive photoreceptor degeneration, leading to impaired dark adaptation, night blindness, gradual visual field damage, and variable final central vision impairment [1]. RP has a complex pathogenesis particularly in its effect on the outer retinal layers [1,2]. Overall, most genes identified to date play important roles in the biochemical pathways of the retinal pigment epithelium and photoreceptor cells [1,3]. However, the importance of the involvement of both the inner retina and the vascular supply has been increasingly recognized in recent years [4,5,6]. In particular, the biomicroscopic aspect of the “pale” optic disc, together with its typical vascular attenuation, suggest the presence of vascular damage. More recently, structural optical coherence tomography (OCT) and OCT angiography (OCTA) have provided further information useful in understanding RP pathogenesis better. Indeed, these techniques are able to investigate retinal microstructural features non-invasively, providing histologically comparable information on the involvement of each retinal layer and the integrity of the retinal vascular network. Regarding RP, previous OCTA studies have shown macular vascular density alterations both in the superficial capillary plexus (SCP) and the deep capillary plexus (DCP) [7,8]. A vascular impairment at the level of the peripapillary network has also been described [9]. Although these findings proved useful in attaining a better understanding of the progression of degenerative phenomena leading to the loss of integrity in both inner and outer retinal layers, the limitations of the current OCTA analysis are related to the fact that the method provides only morphological information. Moreover, no study was specifically designed to investigate the correlations between OCTA vascular findings and clinical and functional outcomes. The application of more advanced quantitative OCTA parameters offers the chance to also explore functional features through OCTA data, thus providing a more accurate patient categorization. The aim of the present study is to quantitatively analyze the retinal vascular plexa at the level of both the macula and the optic nerve in an attempt to identify different vascular patterns, which may influence the final functional outcome.

## 2. Methods

### 2.1. Ophthalmologic Assessment

This study was designed as a pilot, observational, cross-sectional analysis. Consecutive patients affected by RP were recruited in the Retinal Heredodystrophy Unit of the Department of Ophthalmology of San Raffaele Hospital in Milan. This study was approved by the Ethical Committee of the Vita-Salute San Raffaele University in Milan (NET-2016-02363765), in accordance with the Declaration of Helsinki. Signed informed consent was obtained from each patient before the examination. The inclusion criteria were genetically-confirmed diagnosis of RP and age between 18 and 70 years. The following exclusion criteria were adopted: peripapillary atrophy, refractive errors greater than ± 3D, high media opacity, low fixation, any other retinal and/or optic nerve diseases (e.g., diabetic retinopathy, glaucoma), any ophthalmic surgery in the last three months, any systemic conditions potentially affecting the analyses.

The ophthalmologic examination included best corrected visual acuity (BCVA) using the standard Early Treatment Diabetic Retinopathy Study chart, anterior and posterior segment slit-lamp evaluation and Goldmann applanation tonometry for intraocular pressure measurement. OCT and OCTA images were acquired by means of spectral-domain OCT (Spectralis HRA + OCT, Heidelberg Engineering; Heidelberg, Germany; swept-source OCT DRI Topcon Triton, Topcon Corporation; Tokyo, Japan). The OCT acquisition protocol included radial, raster and optic nerve scans, while OCTA analysis included 3 × 3 mm and 4.5 × 4.5 mm acquisitions, centered at the level of both the macula and the optic nerve head. Only high-quality images, in accordance with the Topcon image quality index (≥70) [10], were considered. OCTA images were analyzed with the Topcon full-spectrum amplitude decorrelation angiography algorithm. Moreover, the patients underwent 30-2 Humphrey visual field (VF) (Zeiss Meditec Inc., Dublin, CA, USA), and the data interpretation was based on the visual field index [11]. Each patient contributed with a single eye, which was randomly selected. All data were compared with those obtained from an age- and sex-matched healthy control group.

### 2.2. OCTA Quantitative Analyses

The superficial capillary plexus (SCP), deep capillary plexus (DCP) and choriocapillaris (CC) were automatically segmented in both the macula and the optic nerve head; similarly, the radial peripapillary capillary (RPC) plexus segmentation was automatically obtained at the optic nerve head. Each reconstruction was carefully reviewed by an expert ophthalmologist (MBP) and manually corrected if necessary. Moreover, the same ophthalmologist performed central macular thickness (CMT) and choroidal thickness (CT) measurements on a horizontal structural OCT scan centered on the fovea. Macular parameters will henceforth be referred to by means of the prefix “m”, whereas those concerning the optic nerve head will be prefixed by “n” (e.g., mSCP and nSCP for macular and nerve superficial capillary plexa, respectively).

**Figure 1:**
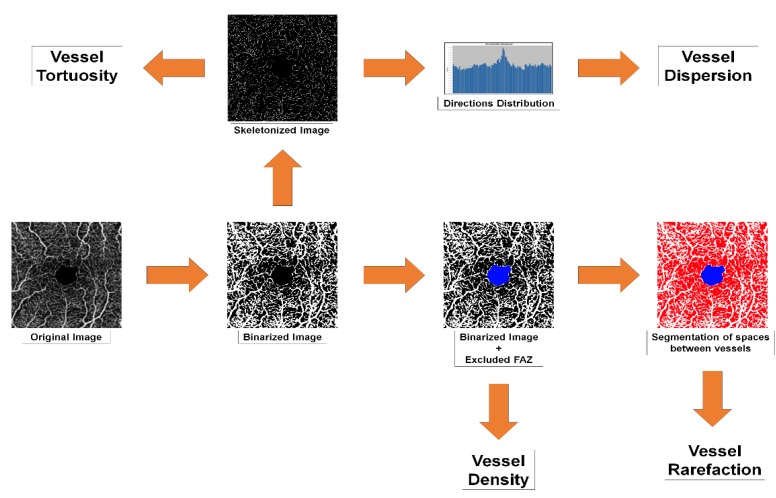
Optical coherence tomography angiography (OCTA) quantitative analyses pipeline. Extensive description is provided in the methods section.

All reconstructions were exported in .tiff format and loaded in ImageJ software (https://imagej.net/Welcome). In-house scripts were built to calculate the following parameters: vessel density (VD), vessel tortuosity (VT), vessel dispersion (VDisp) and vessel rarefaction (VR). The ‘adjust threshold’ tool was used in ImageJ to reduce the noise and to highlight the blood vessels. As CC anatomical features were not compatible with the assessment of VT, VDisp and VR analyses, their analyses were omitted. All images where binarized through a mean threshold to calculate VD. The foveal avascular zone was considered an exclusion criterion. The “skeletonize” function enabled each vessel to be considered as a line. Strahler Analysis was applied the better to pick out each root and to reduce possible bias (https://imagej.net/Strahler_Analysis#Root_Detection) [12]. VT was calculated based on an ImageJ automatic pipeline. The key passages include: binarization, skeletonization, recognition of the branches, then the calculation of the Euclidean distance for each line, which is the final measure of the tortuosity, in accordance with ImageJ pipeline description. The Euclidean distance is the ratio between each line length and the shortest linear distance required to connect the initial and final points. Thus, VT may be considered a quantitative way to describe the path followed by each vessel [13]. VDisp was calculated by assuming that all vessels follow certain directions which can be studied through a Gaussian function. The measure of data dispersion, defined as the standard deviation from the Gaussian distribution, may be understood as the expression of vascular network disorganization [14]. The calculation was done on skeletonized reconstructions by applying the “Directionality” ImageJ function [15], thus extracting data dispersion values. VR was analyzed on binarized images, excluding the foveal avascular zone and the optic nerve head space, using mesh hole analysis, a function available in the DiameterJ plugin [16,17]. This function automatically detected and counted all black pixels of discrete clusters among vessels. VR corresponds to an average representation of how distant vessels are from each other. This approach has already been successfully adopted in other studies to determine porosity within fiber networks [18,19]. Large retinal vessels and the foveal avascular zone (FAZ) area were excluded for the analyses. In particular, large retinal vessels were removed by the projection artifact removal tool provided by the device software. The FAZ area was manually segmented by an expert ophthalmologist (MBP) on SCP and DCP reconstructions. Moreover, the optic nerve head area was manually segmented and excluded from the calculations as well. The complete OCTA quantification pipeline is shown in Figure 1.

Reproducibility and repeatability coefficient were calculated in a separate subset of RP patients, with values ranging from 1.5 to 4.0 (unpublished personal data) [20].

### 2.3. Statistical Analyses

The primary outcome of this study was the characterization of macular and optic nerve head vascular patterns in eyes affected by RP, as examined on OCTA in terms of VD, VT, VDisp, and VR. Secondary outcomes included the identification of correlations among OCTA parameters, BCVA, CMT and VF in an attempt to identify different vascular patterns. We tested possible effects associated with patients’ age and disease duration.

After assessing the homogeneity of the variance and the normality of the distribution of values, we tested the significant differences of the mean values by means of one-way ANOVA test (SPSS; Chicago, IL, USA). We applied Bonferroni correction (*p* value × number of tests) on the resulting *p* values to correct the *p* value for multiple comparisons. Final *p* values lower than 0.05 were considered as statistically significant. Moreover, Kendall’s Tau correlation coefficient measurement was used to perform correlation analysis. 

A Receiver Operating Characteristics (ROC) analysis was performed in order to select OCTA parameters not correlating with patients’ ages, but significantly correlating with BCVA and CMT. For each OCTA parameter, cut-off values were obtained from the mean value of all vascular plexa. From this calculation, we established cut-off values that were independent from possible age effects and able to distinguish two RP sub-groups significantly differing in terms of BCVA, CMT and OCTA parameters. 

## 3. Results 

### 3.1. Main Results

**Figure 2:**
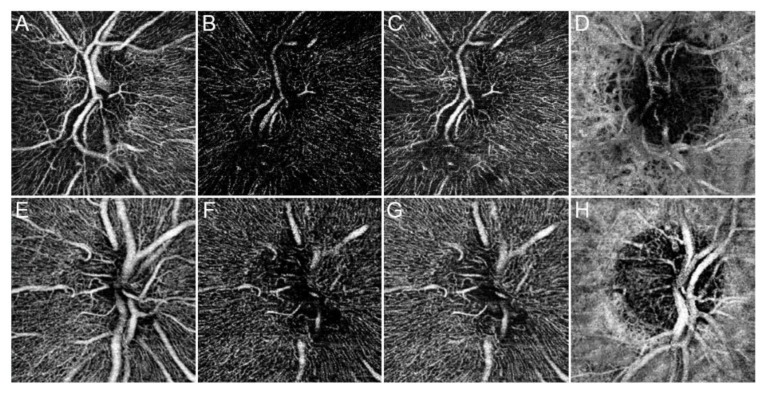
Optic nerve head OCTA in Retinitis Pigmentosa (RP). OCTA reconstructions are shown as follows: superficial capillary plexus (SCP) (**A**), deep capillary plexus (DCP) (**B**), radial peripapillary capillaries (RPCs) (**C**), and choriocapillary (CC) (**D**) for a specimen RP patient and the same plexa for a control subject (**E**–**H**). SCP and CC appeared almost preserved compared with a healthy eye. On the contrary, DCP and RPCs appeared strongly altered in RP. In particular, both not perfused and rarefied regions can be easily qualitatively distinguished.

Overall, 32 eyes of 32 consecutive RP patients (16 males, 50%; mean age 43.2 ± 12.5) were included in the analyses. Disease duration calculated on the basis of the reported onset of the symptoms was 18.2 ± 10.5 years. Likewise, 32 eyes of 32 healthy subjects (16 males, 50%; mean age 45.9 ± 11.4) were included as controls. Mean BCVA, CMT, CT and RNFL were found to be statistically different between patients and controls (all *p* < 0.001) (Table 1). Genetic mutations are reported in Table 2. By way of example, the optic nerve head vascular plexa are shown in Figure 2. 

VD analyses of RP patients revealed statistically significant reductions for all macular and optic nerve head vascular plexa compared with the control group (all *p* < 0.01), the only exceptions being nSCP (*p* = 0.053) and nCC (*p* = 0.081). 

VDisp, VT and VR values were found to be significantly different in all macular and optic nerve head vascular plexa comparing RP patients and controls (all *p* < 0.01). In particular, RP patients showed higher VDisp and VR, and lower VT. The most relevant data are summarized in Table 3.

Correlation analyses revealed several statistically significant links between age, BCVA, OCT and OCTA parameters (Table 4). Interestingly, age correlated significantly with VD and VDisp values. OCTA parameters showed significant correlations with BCVA, RNFL and CMT. In addition, OCTA parameters correlated significantly between each other for each plexus (*p* < 0.01), as follows: VD correlated positively with VT and correlated negatively with VR and VDisp. VT correlated negatively with VR and VDisp, whereas VR correlated positively with VDisp. Moreover, both CMT and RNFL correlated significantly with BCVA. No significant correlations were found between OCTA parameters and VF. Moreover, no significant relationship between OCTA parameters and both genetic tests and disease duration was found. The complete data including both macular and optic nerve head parameters are extensively reported in the Supplementary Materials (Tables S1 and S2).

### 3.2. Cut-Off Analysis to Identify Different RP Subgroups

We attempted to identify separate vascular patterns by singling out OCTA-based cut-offs, taking only VT and VR into consideration. Indeed, VD and VDisp were excluded owing to the presence of age and disease duration effects, which were highlighted by the correlation analyses. The ROC analysis was performed by trying to find two RP subgroups that differed significantly in terms of BCVA. In particular, a VT cut-off of 4.80 was able to distinguish two subgroups with 0.857 sensitivity and 0.909 specificity. Furthermore, the same two RP subgroups can be obtained by a VR cut-off of 0.62, with 0.909 sensitivity and 0.714 specificity.

We compared an RP group characterized by mean VT > 4.80 and mean VR < 0.62 (Group 1), another RP group characterized by mean VT < 4.80 and mean VR > 0.62 (Group 2) and the control group. BCVA and RNFL were found to be significantly lower only in Group 2. CMT was statistically significant among all three groups, with the worst values being identified in Group 2. CT was found not to be statistically significant between Group 1 and Group 2. VD was statistically lower only in Group 2 for all macular and optic nerve head vascular plexa, with the only exceptions being mDCP and nDCP. These were also significantly lower in Group 1, when compared with controls. In addition, VDisp for macular plexa was found to be significantly higher in Group 2, compared with Group 1 and controls, whereas it turned out to differ significantly between the three groups when looking at optic nerve head plexa. The most relevant cut-off values are listed in Table 5, whereas complete data are extensively shown in the Supplementary Materials (Table S3).

## 4. Discussion

The pathogenesis of RP is quite complex and mainly related to genetic alterations at photoreceptors and retinal pigment epithelium cell level, ultimately leading to rod apoptosis. Clinical and OCT data can partially explain the functional impairment [21]. The involvement of the retinal and choroidal vascular network in eyes affected by RP is well known, and it can be clinically identified by the mere detection of retinal vascular attenuation and the waxy pallor of the optic disc. Nevertheless, eyes with ostensibly comparable clinical pictures may show variable visual function, making the correlation among genetic, clinical, and functional findings, challenging. 

Only a few studies specifically focused on the vascular impairment typical of RP using OCTA. In particular, initial investigations showed a generally reduced VD at the level of the SCP and the DCP [7,8,9]. The aim of the present pilot investigation was to extend our knowledge about the macular vascular damage in eyes with RP, applying more advanced OCTA analyses both for the macula and the optic nerve head.

Our results indicate that a straightforward comparison between RP and healthy controls reveals a statistically significant difference in all the parameters. In particular, RNFL, CMT and BCVA turned out to be worst in RP, as well as VD, VT, VR and VDisp. Thus, as expected, RP patients showed both functional and anatomical alterations. In particular, VD and VT are interpretable as related to vascular perfusion reductions, whereas VR and VDisp reflect the impairment of the retinal vascular network [22]. VD reduction has been already reported [7,8,9], and the present study disclosed also VD alterations at the level of the optic nerve head plexa. However, in our opinion, VD is not a reliable parameter because it is influenced by age, as shown by our correlation analysis (Table 3). 

VDisp reflects the degree of disorganization of the retinal vascular network and it can be influenced by a number of possible factors, including loss of vascular microstructural integrity and blood flow impairment but, once again, the correlation analyses revealed the effect of age (Table 3). 

Only VT and VR turned out not to be influenced by age and disease duration and, therefore, we focused on them. 

The statistically significant reduction of VT, corresponding to a vessel rectilinization, may be related to the blood perfusion on the path followed by each vessel. In particular, since the degree of tortuosity of a given vessel is determined by the combination of external wall features and internal perfusion [23,24], we hypothesize that the perfusion reduction in RP contributes to the decrease in VT. In addition, the VR increase may also represent a secondary effect of the progressive perfusion changes occurring in RP, causing an increase in the retinal space among the blood vessels, which become more rarefied.

Wishing to extend our analyses on the basis of the ROC curve, we searched for quantitative cut-offs of VT and VR directly related to BCVA and CMT, which might provide a way to differentiate two RP subgroups on the basis of the vascular response. A subset of eyes (pattern 1) showing a better vascular pattern (higher VT and lower VR values) was characterized by a BCVA similar to that of control subjects, even though both macular and optic nerve head VT and VR were abnormal, indicating the presence of an initial perfusion defect. Interestingly, an increased VDisp was only registered at the level of the optic nerve head plexa, suggesting a potential earlier involvement of the optic nerve head vascular network than the macular one. 

In contrast, another subset of eyes (pattern 2) showed significantly worse imaging-based parameters (CMT, RNFL, quantitative OCTA parameters) and worse BCVA when compared with both pattern 1 subgroup and control subjects. 

Ruling out the age effect, the most reliable OCTA parameters providing information about the severity of vascular impairment in RP patients are VT and VR, which can help differentiate two vascular patterns. However, at present, on the basis of our cross-sectional analysis, it is difficult to tell whether the two vascular patterns are subsequent stages of the same disease [25] or different vascular phenotypes, although the absence of significant age and disease duration effects suggest that the RP subgroups may represent two different RP vascular phenotypes. This aspect could not be determined with the study settings of our pilot investigation and will need further prospective studies.

We are aware that our pilot study has a number of limitations. Firstly, even though our cohort of patients affected by RP is the largest studied by means of OCTA, the number of patients is still insufficient to draw any definitive conclusion, due to the rarity of the disease. In addition, no longitudinal follow-up and no specific genotype–phenotype correlation was attempted, mindful of the initial state of our knowledge of OCTA contribution to the pathophysiology of RP. This study could also be biased by the limitation in defining the onset of the disease, which was based on the patients’ report. The homogeneous response of the vessel density alteration in our patients partly justifies our decision not to complicate the clinical interpretation. Moreover, OCTA analysis is known to be affected by artifacts [26]. In addition, large retinal vessels may have a shadow effect on the vascular plexa, but unfortunately this artifact cannot be removed. On the other hand, it can be assumed that this artifact equally affects controls and RP patients, thus reducing its negative effect on OCTA quantification. Although all possible precautions were taken, OCTA techniques cannot be considered completely reliable in the absence of a clear histological validation. Another limitation is related to the lack of electrophysiological tests, which means further prospective investigations will be required in order to assess their relationship with imaging data. The lack of correlation with the visual field index and genetic tests might be explained by the fact that OCTA is an investigation method confined on the posterior pole, whereas RP changes start and progress from the extreme periphery, with only a later involvement of the posterior pole region. Finally, we have applied new OCTA quantitative parameters to provide a more thorough analysis of the vascular impairment in RP, which need further validation. 

## 5. Conclusions

In essence, the present pilot study indicates that complex vascular alterations are associated with RP, affecting both the macula and the optic nerve head. VT and VR are the most reliable OCTA quantitative parameters to describe the impairment of the vascular patterns in RP patients. The adoption of OCTA quantitative parameters in future clinical interventional settings might have an impact on patient selection in trials and in monitoring the effects of treatments. Further prospective studies with a higher number of patients are warranted to confirm our preliminary data.

## Figures and Tables

**Table 1:** Demographic features of RP patients and healthy controls.

Demographic and Clinical Features		
Parameter	Retinitis Pigmentosa Patients	Controls
Sex (M/F)	16/16	16/16
Age	43.2 ± 12.5	42.8 ± 11.2
BCVA	0.21 ± 0.34	0.0 ± 0.0
CMT	231.49 ± 28.22	301.52 ± 18.55
CT	214.31 ± 41.03	255.85 ± 25.79
RNFL	82.53 ± 19.77	101.21 ± 9.15

The following abbreviations are used: best corrected visual acuity (BCVA), central macular thickness (CMT), choroidal thickness (CT), and retinal nerve fiber layer (RNFL).

**Table 2:** Genetic analysis in Retinitis Pigmentosa.

Genetic Analysis in Retinitis Pigmentosa		
Gene	Number of Patients	%
*ABCA4*	6	18.75
*USH2A*	8	25
*PROM1*	3	9.375
*CYP4V2*	2	6.25
*NR2E3*	1	3.125
*PDE6A*	1	3.125
*RP1L1*	1	3.125
*RPGR*	1	3.125
*CNGA1*	1	3.125
*CNGB1*	1	3.125
*FSCN2*	1	3.125
*BBS1*	1	3.125
*C2ORF71*	1	3.125
*MYO7A*	1	3.125
*CEPB90*	1	3.125
*EYE*	1	3.125
*EYS*	1	3.125

**Table 3:** The most relevant OCTA parameters of RP patients and healthy controls.

OCTA Parameters in Retinitis Pigmentosa						
**Vessel Density Analysis**						
Vascular Plexus	mSCP	*p* Value	mDCP	*p* Value	mCC	*p* Value
RP	0.39 ± 0.02	*p* < 0.01	0.36 ± 0.03	*p* < 0.01	0.49 ± 0.01	*p* < 0.01
Controls	0.41 ± 0.01		0.43 ± 0.01		0.50 ± 0.01	
**Vessel Dispersion Analysis**						
Vascular Plexus	mSCP	*p* Value	mDCP	*p* Value		
RP Patients	24 ± 15	*p* < 0.01	16 ± 12	*p* < 0.01		
Controls	11 ± 4		11 ± 3			
**Vessel Tortuosity Analysis**						
Vascular Plexus	mSCP	*p* Value	mDCP	*p* Value		
RP Patients	4.80 ± 0.29	*p* < 0.01	4.42 ± 0.49	*p* < 0.01		
Controls	7.20 ± 0.31		7.84 ± 0.34			
**Vessel Rarefaction Analysis**						
Vascular Plexus	mSCP	*p* Value	mDCP	*p* Value		
RP Patients	0.66 ± 0.04	*p* < 0.01	0.62 ± 0.03	*p* < 0.01		
Controls	1.80 ± 0.32		1.09 ± 0.20			

The following abbreviations are used: macular superficial capillary plexa (mSCP), macular deep capillary plexus (mDCP), macular choriocapillary (mCC). Extensive data are provided in Table S1.

**Table 4:** Correlation analysis of quantitative parameters. Only the most relevant statistically significant correlations are reported. Complete data are provided in Table S2. Vessel density (VD), vessel tortuosity (VT), vessel dispersion (VDisp), and vessel rarefaction (VR).

Correlation Analysis														
		VD Mean	Vdisp Mean											
AGE	Tau Coeff.	−0.282	0.286											
	*p* value	0.02	0.02											
		CMT	BCVA (logMAR)	VD mSCP	VD mDCP	VD mCC	VD Mean	Vdisp Mean	VT mSCP	VT mDCP	VT Mean	VR mSCP	VR mDCP	VR Mean
RNFL	Tau Coeff.	0.375	−0.548	0.529	0.255	0.44	0.578	−0.368	0.287	0.376	0.448	−0.392	−0.396	−0.481
	*p* value	<0.01	<0.01	<0.01	0.04	<0.01	<0.01	<0.01	0.02	<0.01	<0.01	<0.01	<0.01	<0.01
		BCVA (logMAR)	VD mSCP	VD mDCP	VD mCC	VD Mean	Vdisp mDCP	Vdisp Mean	VT mSCP	VT mDCP	VT Mean	VR mSCP	VR mDCP	VR Mean
CMT	Tau Coeff.	−0.673	0.52	0.313	0.516	0.479	−0.451	−0.447	0.568	0.354	0.576	−0.564	−0.601	−0.625
	*p* value	<0.01	<0.01	<0.01	<0.01	<0.01	<0.01	<0.01	<0.01	<0.01	<0.01	<0.01	<0.01	<0.01
		VD mSCP	VD mDCP	VD mCC	VD Mean	Vdisp mDCP	Vdisp Mean	VT mSCP	VT mDCP	VT Mean	VR mSCP	VR mDCP	VR Mean	
BCVA (logMAR)	Tau Coeff.	−0.443	−0.463	−0.592	−0.573	0.563	0.563	−0.621	−0.463	−0.712	0.645	0.573	0.721	
	*p* value	<0.01	<0.01	<0.01	<0.01	<0.01	<0.01	<0.01	<0.01	<0.01	<0.01	<0.01	<0.01	

**Table 5:** Cut-off analysis in Retinitis Pigmentosa.

OCTA Cut-off Analysis				
Parameter		Mean ± STD	*p* Values	
RNFL	RP1	96 ± 10	RP1 vs. RP2	<0.01
	RP2	62 ± 10	RP1 vs. Controls	0.286
	Controls	101 ± 9	RP2 vs. Controls	<0.01
CMT	RP1	247 ± 21	RP1 vs. RP2	<0.01
	RP2	209 ± 23	RP1 vs. Controls	<0.01
	Controls	302 ± 19	RP2 vs. Controls	<0.01
BCVA (logMAR)	RP1	0.01 ± 0.04	RP1 vs. RP2	<0.01
	RP2	0.49 ± 0.38	RP1 vs. Controls	0.94
	Controls	0 ± 0	RP2 vs. Controls	<0.01
VD mSCP	RP1	0.41 ± 0.02	RP1 vs. RP2	<0.01
	RP2	0.38 ± 0.01	RP1 vs. Controls	0.976
	Controls	0.41 ± 0.01	RP2 vs. Controls	<0.01
VD mDCP	RP1	0.37 ± 0.03	RP1 vs. RP2	<0.01
	RP2	0.35 ± 0.02	RP1 vs. Controls	<0.01
	Controls	0.43 ± 0.01	RP2 vs. Controls	<0.01
VD mCC	RP1	0.50 ± 0.02	RP1 vs. RP2	<0.01
	RP2	0.47 ± 0.01	RP1 vs. Controls	0.768
	Controls	0.50 ± 0.01	RP2 vs. Controls	<0.01
Vdisp mSCP	RP1	12.76 ± 3.71	RP1 vs. RP2	<0.01
	RP2	21.42 ± 15.77	RP1 vs. Controls	0.92
	Controls	10.72 ± 4.15	RP2 vs. Controls	<0.01
Vdisp mDCP	RP1	13.66 ± 4.51	RP1 vs. RP2	<0.01
	RP2	34.75 ± 9.43	RP1 vs. Controls	0.53
	Controls	11.45 ± 3.48	RP2 vs. Controls	<0.01
VT mSCP	RP1	5.16 ± 0.34	RP1 vs. RP2	<0.01
	RP2	4.56 ± 0.15	RP1 vs. Controls	<0.01
	Controls	7.20 ± 0.31	RP2 vs. Controls	<0.01
VT mDCP	RP1	4.86 ± 0.29	RP1 vs. RP2	<0.01
	RP2	4.23 ± 0.35	RP1 vs. Controls	<0.01
	Controls	7.84 ± 0.34	RP2 vs. Controls	<0.01
VR mSCP	RP1	0.62 ± 0.03	RP1 vs. RP2	<0.01
	RP2	0.70 ± 0.02	RP1 vs. Controls	<0.01
	Controls	0.41 ± 0.01	RP2 vs. Controls	<0.01
VR mDCP	RP1	0.59 ± 0.03	RP1 vs. RP2	<0.01
	RP2	0.65 ± 0.02	RP1 vs. Controls	<0.01
	Controls	0.43 ± 0.01	RP2 vs. Controls	<0.01

Retinitis Pigmentosa (RP)1 was defined with mean VT > 4.80 and mean VR < 0.62, whereas RP2 was defined with mean VT < 4.80 and mean VR > 0.62. Only the most relevant data are reported. Complete data are extensively shown in Table S3. Central macular thickness (CMT) and retinal nerve fiber layer (RNFL).

## Supplementary Materials

The following are available online at https://www.mdpi.com/2077-0383/8/9/1425/s1, Table S1: Complete OCTA parameters of RP patients and healthy controls; Table S2: Correlation analysis of quantitative parameters; Table S3: Complete cutoff analysis in Retinitis Pigmentosa.
