# Supplementary material for: Vascular Patterns in Retinitis Pigmentosa on Swept-Source Optical Coherence Tomography Angiography
Source: J Clin Med. 2019 Sep 10;8(9):1425. doi: 10.3390/jcm8091425 (PMC6780333; doi:10.3390/jcm8091425)
Supplement: Supplementary file 1 [file jcm-08-01425-s001.pdf]

Journal of Clinical Medicine

MDPI

Supplementary Materials

Table S1: Complete OCTA parameters of RP patients and healthy controls. The following abbreviations are used: superficial capillary plexus (SCP), deep capillary plexus (DCP) and choriocapillary (CC). Macular and optic nerve head plexa are distinguished by “m” and “n”, respectively.

OCTA Parameters in Retinitis Pigmentosa

Vessel Density Analysis

Vascular Plexus	mSCP	*p* Value	mDCP	*p* Value	mCC	*p* Value	RPC	*p* Value	nSCP	*p* Value	nDCP	*p* Value	nCC	*p* Value
**RP**	0.39 ± 0.02	$p < 0.01$	0.36 ± 0.03	$p < 0.01$	0.49 ± 0.01	$p < 0.01$	0.40 ± 0.02	$p < 0.01$	0.42 ± 0.03	$p > 0.05$	0.30 ± 0.02	$p < 0.01$	0.54 ± 0.05	$p > 0.05$
**Controls**	0.41 ± 0.01		0.43 ± 0.01		0.50 ± 0.01		0.45 ± 0.01		0.43 ± 0.01		0.39 ± 0.02		0.54 ± 0.03	

Vessel Dispersion Analysis

Vascular Plexus	mSCP	*p* Value	mDCP	*p* Value	RPC	*p* Value	nSCP	*p* Value	nDCP	*p* Value
**RP Patients**	24 ± 15	$p < 0.01$	16 ± 12	$p < 0.01$	37 ± 15	$p < 0.01$	27 ± 14	$p < 0.01$	35 ± 13	$p < 0.01$
**Controls**	11 ± 4		11 ± 3		11 ± 4		10 ± 3		10 ± 3	

Vessel Tortuosity Analysis

Vascular Plexus	mSCP	*p* Value	mDCP	*p* Value	RPC	*p* Value	nSCP	*p* Value	nDCP	*p* Value
**RP Patients**	4.80 ± 0.29	$p < 0.01$	4.42 ± 0.49	$p < 0.01$	5.11 ± 0.39	$p < 0.01$	5.27 ± 0.26	$p < 0.01$	4.01 ± 0.25	$p < 0.01$
**Controls**	7.20 ± 0.31		7.84 ± 0.34		7.73 ± 0.30		8.42 ± 0.33		7.06 ± 0.25	

Vessel Rarefaction Analysis

Vascular Plexus	mSCP	*p* Value	mDCP	*p* Value	RPC	*p* Value	nSCP	*p* Value	nDCP	*p* Value
**RP Patients**	0.66 ± 0.04	$p < 0.01$	0.62 ± 0.03	$p < 0.01$	0.64 ± 0.08	$p < 0.01$	0.67 ± 0.05	$p < 0.01$	0.48 ± 0.04	$p < 0.01$
**Controls**	1.80 ± 0.32		1.09 ± 0.20		1.15 ± 0.22		1.52 ± 0.16		0.99 ± 0.07	

Table S2: Correlation analysis of quantitative parameters. All statistically significant correlations are reported. The following abbreviations are used: superficial capillary plexus (SCP), deep capillary plexus (DCP) and choriocapillary (CC). Macular and optic nerve head plexa are distinguished by “m” and “n”, respectively.

Correlation Analysis

		VD Mean	Vdisp Mean
AGE	Tau Coeff.	−0.282	0.286
	p value	0.02	0.02

		CMT	BCVA (logMAR)	VD mSCP	VD mDCP	VD mCC	VD RPC	VD nSCP	VD nDCP	VD nCC	VD Mean	Vdisp Mean	VT mSCP	VT mDCP	VT RPC	VT nSCP	VT nDCP	VT Mean	VR mSCP	VR mDCP	VR RPC	VR nSCP	VR nDCP	VR Mean
RNFL	Tau Coeff.	0.375	−0.548	0.529	0.255	0.44	0.695	0.588	0.447	0.424	0.578	−0.368	0.287	0.376	0.477	0.251	0.36	0.448	−0.392	−0.396	−0.505	−0.44	−0.291	−0.481
	p value	<0.01	<0.01	<0.01	0.04	<0.01	<0.01	<0.01	<0.01	<0.01	<0.01	<0.01	0.02	<0.01	<0.01	0.04	<0.01	<0.01	<0.01	<0.01	<0.01	<0.01	0.02	<0.01

		BCVA (logMAR)	VD mSCP	VD mDCP	VD mCC	VD RPC	VD nSCP	VD nDCP	VD nCC	VD Mean	Vdisp mDCP	Vdisp RPC	Vdisp nSCP	Vdisp nDCP	Vdisp Mean	VT mSCP	VT mDCP	VT RPC	VT nSCP	VT nDCP	VT Mean	VR mSCP	VR mDCP	VR RPC	VR nSCP	VR nDCP	VR Mean
CMT	Tau Coeff.	−0.673	0.52	0.313	0.516	0.423	0.466	0.352	0.415	0.479	−0.451	−0.289	−0.366	−0.281	−0.447	0.568	0.354	0.556	0.633	0.455	0.576	−0.564	−0.601	−0.52	−0.495	−0.52	−0.625
	p value	<0.01	<0.01	<0.01	<0.01	<0.01	<0.01	<0.01	<0.01	<0.01	<0.01	0.02	<0.01	0.02	<0.01	<0.01	<0.01	<0.01	<0.01	<0.01	<0.01	<0.01	<0.01	<0.01	<0.01	<0.01	<0.01

		VD mSCP	VD mDCP	VD mCC	VD RPC	VD nSCP	VD nDCP	VD nCC	VD Mean	Vdisp mDCP	Vdisp RPC	Vdisp nSCP	Vdisp nDCP	Vdisp Mean	VT mSCP	VT mDCP	VT RPC	VT nSCP	VT nDCP	VT Mean	VR mSCP	VR mDCP	VR RPC	VR nSCP	VR nDCP	VR Mean
BCVA (logMAR)	Tau Coeff.	−0.443	−0.463	−0.592	−0.506	−0.461	−0.278	−0.558	−0.573	0.563	0.371	0.429	0.371	0.563	−0.621	−0.463	−0.568	−0.64	−0.52	−0.712	0.645	0.573	0.602	0.592	0.472	0.721
	p value	<0.01	<0.01	<0.01	<0.01	<0.01	0.04	<0.01	<0.01	<0.01	<0.01	<0.01	<0.01	<0.01	<0.01	<0.01	<0.01	<0.01	<0.01	<0.01	<0.01	<0.01	<0.01	<0.01	<0.01	<0.01

Table S3: Complete cutoff analysis in Retinitis Pigmentosa. GROUP1 was defined with mean VT > 4.80 and mean VR < 0.62. whereas GROUP2 was defined with mean VT < 4.80 and mean VR > 0.62. The following abbreviations are used: superficial capillary plexus (SCP), deep capillary plexus (DCP) and choriocapillary (CC). Macular and optic nerve head plexa are distinguished by "m" and "n", respectively.

OCTA Cutoff Analysis				
Parameter		Mean ± STD	*p* values	
RNFL	RP1	96 ± 10	RP1 vs RP2	<0.01
	RP2	62 ± 10	RP1 vs Controls	0.286
	Controls	101 ± 9	RP2 vs Controls	<0.01
CMT	RP1	247 ± 21	RP1 vs RP2	<0.01
	RP2	209 ± 23	RP1 vs Controls	<0.01
	Controls	302 ± 19	RP2 vs Controls	<0.01
BCVA (logMAR)	RP1	0.01 ± 0.04	RP1 vs RP2	<0.01
	RP2	0.49 ± 0.38	RP1 vs Controls	0.94
	Controls	0 ± 0	RP2 vs Controls	<0.01
VD mSCP	RP1	0.41 ± 0.02	RP1 vs RP2	<0.01
	RP2	0.38 ± 0.01	RP1 vs Controls	0.976
	Controls	0.41 ± 0.01	RP2 vs Controls	<0.01
VD mDCP	RP1	0.37 ± 0.03	RP1 vs RP2	<0.01
	RP2	0.35 ± 0.02	RP1 vs Controls	<0.01
	Controls	0.43 ± 0.01	RP2 vs Controls	<0.01
VD mCC	RP1	0.50 ± 0.02	RP1 vs RP2	<0.01
	RP2	0.47 ± 0.01	RP1 vs Controls	0.768
	Controls	0.50 ± 0.01	RP2 vs Controls	<0.01
VD RPC	RP1	0.45 ± 0.01	RP1 vs RP2	<0.01
	RP2	0.38 ± 0.02	RP1 vs Controls	0.604
	Controls	0.45 ± 0.01	RP2 vs Controls	<0.01
VD nSCP	RP1	0.43 ± 0.01	RP1 vs RP2	<0.01
	RP2	0.40 ± 0.02	RP1 vs Controls	0.582
	Controls	0.43 ± 0.01	RP2 vs Controls	<0.01
VD nDCP	RP1	0.31 ± 0.01	RP1 vs RP2	<0.01
	RP2	0.29 ± 0.03	RP1 vs Controls	<0.01
	Controls	0.40 ± 0.02	RP2 vs Controls	<0.01
VD nCC	RP1	0.53 ± 0.02	RP1 vs RP2	<0.01
	RP2	0.48 ± 0.05	RP1 vs Controls	0.88
	Controls	0.54 ± 0.03	RP2 vs Controls	<0.01
VD Mean	RP1	0.43 ± 0.01	RP1 vs RP2	0.06
	RP2	0.39 ± 0.01	RP1 vs Controls	<0.01
	Controls	0.45 ± 0.01	RP2 vs Controls	<0.01
Vdisp mSCP	RP1	12.76 ± 3.71	RP1 vs RP2	<0.01
	RP2	21.42 ± 15.77	RP1 vs Controls	0.92

	Controls	10.72 ± 4.15	RP2 vs Controls	<0.01
Vdisp mDCP	RP1	13.66 ± 4.51	RP1 vs RP2	<0.01
	RP2	34.75 ± 9.43	RP1 vs Controls	0.53
	Controls	11.45 ± 3.48	RP2 vs Controls	<0.01
Vdisp RPC	RP1	24.11 ± 6.33	RP1 vs RP2	<0.01
	RP2	38.23 ± 16.41	RP1 vs Controls	<0.01
	Controls	10.61 ± 3.70	RP2 vs Controls	<0.01
Vdisp nSCP	RP1	20.47 ± 7.90	RP1 vs RP2	<0.01
	RP2	35.15 ± 11.14	RP1 vs Controls	<0.01
	Controls	10.35 ± 2.88	RP2 vs Controls	<0.01
Vdisp nDCP	RP1	25.58 ± 9.75	RP1 vs RP2	<0.01
	RP2	41.12 ± 11.27	RP1 vs Controls	<0.01
	Controls	10.37 ± 3.36	RP2 vs Controls	<0.01
Vdisp Mean	RP1	19.63 ± 4.40	RP1 vs RP2	<0.01
	RP2	30.13 ± 5.47	RP1 vs Controls	<0.01
	Controls	10.70 ± 1.35	RP2 vs Controls	<0.01
VT mSCP	RP1	5.16 ± 0.34	RP1 vs RP2	<0.01
	RP2	4.56 ± 0.15	RP1 vs Controls	<0.01
	Controls	7.20 ± 0.31	RP2 vs Controls	<0.01
VT mDCP	RP1	4.86 ± 0.29	RP1 vs RP2	<0.01
	RP2	4.23 ± 0.35	RP1 vs Controls	<0.01
	Controls	7.84 ± 0.34	RP2 vs Controls	<0.01
VT RPC	RP1	5.48 ± 0.40	RP1 vs RP2	<0.01
	RP2	4.87 ± 0.26	RP1 vs Controls	<0.01
	Controls	7.73 ± 0.30	RP2 vs Controls	<0.01
VT nSCP	RP1	5.55 ± 0.32	RP1 vs RP2	<0.01
	RP2	4.97 ± 0.36	RP1 vs Controls	<0.01
	Controls	8.42 ± 0.33	RP2 vs Controls	<0.01
VT nDCP	RP1	4.50 ± 0.45	RP1 vs RP2	<0.01
	RP2	3.92 ± 0.27	RP1 vs Controls	<0.01
	Controls	7.06 ± 0.25	RP2 vs Controls	<0.01
VT Mean	RP1	5.11 ± 0.25	RP1 vs RP2	<0.01
	RP2	4.51 ± 0.12	RP1 vs Controls	<0.01
	Controls	7.65 ± 0.23	RP2 vs Controls	<0.01
VR mSCP	RP1	0.62 ± 0.03	RP1 vs RP2	<0.01
	RP2	0.70 ± 0.02	RP1 vs Controls	<0.01
	Controls	0.41 ± 0.01	RP2 vs Controls	<0.01
VR mDCP	RP1	0.59 ± 0.03	RP1 vs RP2	<0.01
	RP2	0.65 ± 0.02	RP1 vs Controls	<0.01
	Controls	0.43 ± 0.01	RP2 vs Controls	<0.01
VR RPC	RP1	0.59 ± 0.07	RP1 vs RP2	<0.01
	RP2	0.69 ± 0.03	RP1 vs Controls	<0.01

	Controls	0.47 ± 0.01	RP2 vs Controls	<0.01
VR nSCP	RP1	0.61 ± 0.04	RP1 vs RP2	<0.01
	RP2	0.70 ± 0.04	RP1 vs Controls	<0.01
	Controls	0.46 ± 0.01	RP2 vs Controls	<0.01
VR nDCP	RP1	0.48 ± 0.06	RP1 vs RP2	<0.01
	RP2	0.54 ± 0.05	RP1 vs Controls	<0.01
	Controls	0.42 ± 0.01	RP2 vs Controls	<0.01
VR Mean	RP1	0.58 ± 0.04	RP1 vs RP2	<0.01
	RP2	0.66 ± 0.01	RP1 vs Controls	<0.01
	Controls	0.44 ± 0.01	RP2 vs Controls	<0.01
